# On Fatigue of the Voice in Its Relations to the Mode of Respiration

**Published:** 1855-07

**Authors:** L. Mandl


### ARTICLE X.

### *On Fatigue of the Voice in its Relations to the Mode of Respiration.* By Dr. L. Mandl.

The physician is occasionally consulted by singers and public speakers for a peculiar weakness of voice which the most minute examination does not justify us in referring to any of the organic causes described in pathological works. Still, when such persons have sung or spoken for some time, their voice is observed to grow weak, and to become masked, hoarse, and shrill. This change, at first slight and transient, subsequently occurs with great facility on the least exercise of the organs; finally, the voice is completely lost; it becomes, as it is called, shattered or cracked.

Uncertain at first as to the cause of this affection, I afterwards thought that my attention should be directed to the action of the larynx itself, and I inquired to what extent an excessive and fatiguing expenditure of strength in the emission of the voice, might contribute to the origin of this enfeeblement. The investigation of this question soon led me to consider the various modes of respiration to which professors of singing attach so much importance. Many of them assert that respiration performed with the upper part of the thorax breaks the voice, and experience seems to support these views. We must endeavor then to demonstrate by anatomical and physiological researches, the relation which may exist between the mode of respiration and the expenditure of the forces necessary to set in action the agents in the production of the voice. This expenditure is the measure of the fatigue.

The emission of the voice takes place during expiration, which must be maintained so long as a musical phrase or sentence continues. The expiratory agents which expel the air in order to the production of sound, are consequently engaged in a struggle with those of inspiration, whose efforts are directed to its retention, in order to prolong the expiration.

The contest which is thus established between the agents of inspiration and those of expiration, and which we call the vocal struggle, varies according to the type of the respiration; the more considerable it is, the greater is the resulting fatigue. In order to be able to appreciate and determine with exactness the degree of fatigue connected with this struggle, it is necessary first to examine the play of the muscles in all points engaged in respiration, whatever be the mode in which this function is accomplished.

Respiration may take place after three different types, the diaphragmatic or abdominal, the lateral (costo-inferior,) and the clavicular (costo-superior of MM. Beau and Massiat.) In the first, we observe the abdominal parietes alternately to rise and subside principally through the action of the diaphragm; in the second, it is the lower ribs; in the last, it is the upper ribs, and especially the first, and with it the sternum and clavicle, which are displaced.

The contest between inspiration and expiration, that is to say, the vocal struggle, and consequently also the resulting fatigue, is least in abdominal respiration, because then only a small number of muscles (chiefly the diaphragm) are set in action, because it is only the soft and mobile viscera of the abdominal cavity which undergo displacement; because during inspiration the larynx remains in its normal position; because the glottis undergoes neither remarkable enlargement nor contraction; and because the chordæ vocales are neither relaxed nor extended in an appreciable manner. The expiration necessary to the modulation of the sound consequently finds the principal organs in their natural position and degree of tension. The displacement of the larynx, the narrowing of the glottis, the tension of the chordæ vocales, the dilatation of the lungs, everything necessary to the production of sound, may, consequently, be effected without resistance, without remarkable efforts, and therefore without fatigue.

In clavicular respiration, on the contrary, the vocal struggle and its attending fatigue are very considerable; because many muscles are engaged in inspiration and expiration; because the fixed and resisting parts composing the upper portion of the thorax must be displaced; because the larynx is strongly depressed when the first rib and sternum are elevated in clavicular inspiration. In abdominal inspiration, these two bones are motionless; consequently the larynx does not alter its situation.

This depression is effected by means of the sterno-thyroid and sterno-hyoid muscles, attached to the first rib and the sternum. In fact, in clavicular respiration the first rib and sternum are elevated with the aid of the scaleni, the sterno-mastoid muscles, &c.; all the muscles attached to the first rib and the sternum are required to effect this displacement; consequently, the sterno-thyroid and sterno-hyoid participate in the action; but the second points of attachment of these two muscles are to the thyroid cartilage and the os hyoids which are movable, and therefore cannot serve as fixed points. Hence the larynx is necessarily depressed, the glottis widened, and the chordæ vocales relaxed during inspiration, and because during the expiration necessary to the modulation of the sound, the larynx, glottis, and chordæ vocales must be in diametrically opposite states. All these movements are so linked to one another, that the simple inspection of the clavicle and shoulders enables us to infer the position of the larynx.

These opposite tractions exercised on the larynx during singing when clavicular respiration is adopted, render the emission of the voice more difficult, more fatiguing, and less harmonious.

The vigorous effort, the tumefaction of the neck, the distension of the jugular veins, the turning of the head, the noisy inspiration, form the usual accompaniments of this faulty respiration; it may even occasion, in time, in the muscles concerned, an excessive sensibility and spasmodic contractions; the twitchings in the mammary region, the sudden attacks of hoarseness are thus frequently explained. This pathological state may produce in the proper muscles of the larynx atrophy more or less complete; with loss of contractility and consecutive aphonia.

Lateral respiration is always combined in deep inspirations with the clavicular or abdominal type, of the inconveniences and advantages of which it will consequently partake.

The physician ought, therefore, resting on anatomical and physiological reasons, to insist that the clavicular type of respiration be banished from instruction and from the practice of the singer. The experience of artists, and the system of instruction of some of our first masters, have long since decided in favor of this view.

I am, therefore, far from pretending to be the first to point out the evils of clavicular respiration; my object is solely to demonstrate by scientific proofs, the forced and invariable consequences of certain respiratory movements, and to explain by anatomical arguments the opinion of some artists.

Moreover, nature furnishes a striking proof of the truth of the preceding remarks. Clavicular respiration, in fact, is impossible in birds who are received as models in song; in them the abdominal parietes alone are dilated during inspiration, while the thorax remains motionless in the entire of its upper portion. The lungs attached posteriorly push the intestines before them with the aid of numerous äerial sacs which discharge the functions of the diaphragm; but clavicular respiration is impossible, for in birds, the sterno-mastoid and trapezoid muscles are wanting, as well as, according to M. Bernard, the external branch of the spinal. In this we have, we may say, a lesson given by nature itself to artists.—*Gaz. Med. de Paris,* March 24, 1855.

